# Development of plasma ghrelin level as a novel marker for gastric mucosal atrophy after *Helicobacter pylori* eradication

**DOI:** 10.1080/07853890.2021.2024875

**Published:** 2022-01-10

**Authors:** Hideki Mori, Hidekazu Suzuki, Juntaro Matsuzaki, Kaori Kameyama, Koji Igarashi, Tatsuhiro Masaoka, Takanori Kanai

**Affiliations:** a Translational Research Center for Gastrointestinal Diseases (TARGID), University of Leuven, Leuven, Belgium; b Division of Gastroenterology and Hepatology, Department of Internal Medicine, Keio University School of Medicine, Tokyo, Japan; c Division of Gastroenterology and Hepatology, Department of Internal Medicine, Tokai University School of Medicine, Isehara, Japan; d Division of Pharmacotherapeutics, Keio University Faculty of Pharmacy, Tokyo, Japan; e Department of Diagnostic Pathology, School of Medicine, Showa University, Yokohama Northern Hospital, Kanagawa, Japan; f Bioscience Division, TOSOH Corporation, Kanagawa, Japan; g Department of Gastroenterology and Hepatology, International University of Health and Welfare, Mita Hospital, Tokyo, Japan

**Keywords:** Ghrelin, pepsinogen, gastric cancer, atrophic gastritis, intestinal metaplasia

## Abstract

**Background and aim:**

The severity of atrophic gastritis is significantly associated with the risk of gastric cancer. Although the current gold standard for assessing the gastric cancer risk is esophagogastroduodenoscopy with a pathological examination, the development of less-invasive biomarkers is warranted for efficient risk stratification of gastric cancer. Serum pepsinogens (PGs) are biomarkers used to predict the extent of gastric mucosal atrophy; however, they are not an accurate reflection of gastric mucosal atrophy after *Helicobacter pylori* eradication. The present study was conducted to investigate the usefulness of plasma ghrelin levels as a marker for gastric mucosal atrophy, and as a risk stratification marker for gastric cancer, even after *H. pylori* eradication.

**Methods:**

Patients who received *H. pylori* eradication treatment were enrolled in the study. The severity of gastric mucosal atrophy was evaluated both endoscopically and histologically. Serum pepsinogen and plasma ghrelin levels were measured before and at 1, 12, 24, and 48 weeks after treatment. The study was approved by the Research Ethics Committee of the Keio University School of Medicine (no. 20140102; 8 July 2014).

**Results:**

Eighteen patients completed the study protocol. Total and acyl plasma ghrelin levels demonstrated no significant change from before treatment to 48 weeks after eradication; however, there was a significant difference between open-type and closed-type atrophic gastritis. The PG I/II ratio increased significantly from 48 weeks after *H. pylori* eradication. The severity of the histological intestinal metaplasia scores correlated inversely with plasma total ghrelin levels from before to 48 weeks after *H. pylori* eradication.

**Conclusion:**

Plasma levels of ghrelin correlate well with the level of gastric mucosal atrophy, even after *H. pylori* eradication.

**KEY MESSAGES**
- Ghrelin plasma levels are associated with the progression of endoscopic atrophic gastritis, even at 48 weeks after *H. pylori* eradication.
- Ghrelin plasma levels are also associated with increased severity of histological intestinal metaplasia 48 weeks after *H. pylori* eradication.
- Pepsinogen I/II ratios increased immediately after *H. pylori* eradication and are inappropriate for assessing atrophic gastritis after *H. pylori* eradication.

## Introduction

*Helicobacter pylori* causes gastric cancer by directly injecting the oncoprotein CagA into gastric epithelial cells and inducing carcinogenesis; therefore, eradicating *H. pylori* prevents carcinogenesis. However, there is a risk of developing gastric cancer even after curing *H. pylori* infection and after treatment of gastric inflammation [1–4]. A longitudinal cohort study showed that the more extensive atrophic gastritis at the time of *H. pylori* eradication, the greater the incidence of gastric cancer after eradication [5]. The only current tool for assessing the extent of gastric mucosal atrophy is direct endoscopic evaluation; however, endoscopy is an invasive procedure that can cause severe discomfort for some patients. Population-based gastric cancer screening has been conducted under a governmental subsidy in Japan, but Japanese gastric cancer screening with X-ray photofluorography or endoscopy has been criticized for its low uptake rate [6]. Therefore, there is a need to develop a non-invasive modality that can assess the degree of gastric mucosal atrophy, regardless of the presence or absence of *H. pylori* infection.

Serum pepsinogens (PGs) are biomarkers for predicting the status and extent of gastric mucosal atrophy [7]. The combination of the PG I concentration and the PG I/II ratio named the ABC method, is used for gastric cancer screening in East Asian countries [8,9]. However, both PG I and PG II are altered by *H. pylori* eradication, which increases the PG I/II ratio and makes it difficult to accurately assess the risk of gastric cancer in post-eradication subjects [10]. Ghrelin is released by gastric P/D1-cells in humans. In addition to stimulating growth hormone release and food intake, ghrelin stimulates gastric acid secretion and gastrointestinal motility, as well as modulating energy balance, taste sensation, stress, anxiety, glucose metabolism, and cardiovascular effects [11–13]. Ghrelin plasma levels are associated with the status of *H. pylori* infection [14]. Indeed, we reported previously that plasma levels of ghrelin decrease with the increasing extent of gastric mucosal atrophy [15,16]. Based on these data, it is natural to assume that damage to the gastric mucosa caused by *H. pylori* infection results in a decrease in P/D1-cells, followed by a decrease in plasma ghrelin. Just as the atrophy of the gastric mucosa does not recover immediately after eradication, it is thought that plasma ghrelin does not recover easily because P/D1-cells take time to recover. To date, no prospective studies have examined changes in plasma ghrelin concentrations over time following *H. pylori* eradication. Therefore, we conducted this pilot study to evaluate the diagnostic utility of plasma ghrelin levels (compared with PG levels) for predicting the severity of gastric mucosal atrophy after *H. pylori* eradication.

In recent years, *H. pylori*-associated dyspepsia, a condition that improves after the eradication of *H. pylori*, has attracted attention, suggesting that eradication of *H. pylori* may improve gastrointestinal symptoms and nutritional status [17–20]. However, *H. pylori* eradication may alter the gastric and intestinal microflora [21–24]. The present study also investigated the relationship between *H. pylori* infection and lipid metabolism, carbohydrate metabolism, and related hormones.

## Materials and methods

### Study population

This was a prospective, observational study conducted at Keio University Hospital (Tokyo, Japan). The study was approved by the Research Ethics Committee of the Keio University School of Medicine (no. 20140102; 8 July 2014) and registered with the UMIN Clinical Trials Registry (UMIN 000014496 [http://www.umin.ac.jp/ctr/]). From March to November 2015, patients who received *H. pylori* eradication treatment were enrolled after providing written informed consent.

### Study design

All enrolled patients had *H. pylori* infection (confirmed using the culture method) before eradication. The extent of gastric mucosal atrophy was assessed endoscopically based on the Kimura–Takemoto classification, which comprises two main types: closed-type (C-type, C1–C3) and open-type (O-type, O1–O3) [25,26]. The severity of histological gastric mucosal atrophy and intestinal metaplasia was assessed using the operative link for gastritis assessment (OLGA) and operative link for gastric intestinal metaplasia assessment (OLGIM) staging systems [27,28]. Serum high-density lipoprotein cholesterol (HDL-C), low-density lipoprotein cholesterol (LDL-C), triglycerides (TG), haemoglobin A1c (HbA1c), glucose, adiponectin, serum PG I and II, serum *H. pylori* immunoglobulin G (HP-IgG), and plasma acyl and des-acyl ghrelin levels were measured before and at 1, 12, 24, and 48 weeks after *H. pylori* eradication treatment. Twelve weeks after the end of eradication therapy, successful eradication was confirmed using a [13C] urea breath test (UBT) or the *H. pylori* stool antigen test [29,30]. The cut-off value for a negative UBT was <2.5‰ [4]. Patients in whom eradication failed were excluded from the analysis.

### Plasma ghrelin assay

Blood samples were collected between 8 and 10 am after an overnight fast. Samples were transferred to a chilled tube containing aprotinin and ethylenediaminetetraacetic acid (EDTA) and then centrifuged immediately (3000 rpm for 15 min). One millilitre of plasma was added to a serum tube containing 100 µl of 1 N HCl and stored at −20 °C until assayed. Acyl and des-acyl plasma ghrelin concentrations were measured using an automated enzyme immunoassay analyzer AIA system (TOSOH Corporation, Tokyo, Japan). The AIA system includes automated specimen dispensation, incubation of the reaction cup, bound/free washing procedure, 4-methylumbelliferyl phosphate substrate dispensation, fluorometric detection, and a result report.

A two-site sandwich immunoassay specific for acyl ghrelin and des-acyl ghrelin was used (TOSOH Corporation). In brief, plasma levels of both acyl and des-acyl plasma ghrelin assays were measured using two independent antibodies: one antibody in the acyl ghrelin assay binds to the N-terminal region, including serine (amino acid 3) with an acyl chain, and the other binds to the C-terminal region; and one antibody in the des-acyl ghrelin assay binds to the N-terminal region, including serine without an acyl chain, and the other binds to the C-terminal region.

### Outcomes

The main outcome measure was the clinical utility of the plasma ghrelin level and PG I/II ratio compared with that of a marker of endoscopic atrophic gastritis (the Kimura–Takemoto classification) and markers of histological atrophic gastritis (OLGA and OLGIM), even after *H. pylori* eradication. The secondary outcome measure was the relationship between *H. pylori* eradication and lipid metabolism, carbohydrate metabolism, and related hormones.

### Statistical analysis

Comparisons of the patients’ demographic characteristics were conducted using Fisher’s exact test and Student’s *t*-test, as appropriate. Comparisons between the endoscopic/histological gastritis grade and plasma ghrelin or PG levels were conducted using Pearson’s correlation analysis. Trends in blood markers and the type of gastric atrophy from before to 48 weeks after eradication were analyzed by two-way repeated-measures ANOVA. Statistical analyses were performed using SPSS 25 for Windows (SPSS Inc., Chicago, IL, USA). Data are expressed as the mean ± standard deviation. Statistical significance was defined as *p* < .05; *p*-values between .05 and .1 were defined as marginally significant.

## Results

### Patient characteristics

A total of 27 patients were enrolled in the study. Of these, six were excluded due to a negative culture of *H. pylori* before eradication, and three were excluded due to eradication failure. Finally, 18 patients (10 men and eight women; mean age, 59.3 ± 11.7 years) completed the protocol (Table 1). Evaluation of gastric mucosal atrophy before eradication by the endoscopic Kimura–Takemoto classification revealed 12 cases of closed-type (C-1: *N* = 4, C-2: *N* = 5, C-3: *N* = 3) and six cases of open-type (O-1: *N* = 4, O-2: *N* = 1, O-3: *N* = 1) atrophic gastritis. Total and acyl plasma ghrelin levels of patients with closed-type atrophic gastritis were higher than those of patients with open-type atrophic gastritis before treatment (total ghrelin: mean, 72.3 ± 30.8 *vs.* 31.7 ± 14.4 fmol/ml, respectively, *p* < .01; acyl ghrelin: mean 15.0 ± 11.1 *vs.* 5.2 ± 3.2 fmol/ml, respectively, *p* = .05).

**Table 1.** Patient demographics.

	Total	Closed-type gastritis	Open-type gastritis	*p*-Value
	*n* = 18	*n* = 12	*n* = 6	
Mean age (years [mean ± *SD*])	59.3 ± 11.7	57.2 ± 8.6	63.5 ± 16.5	.29^†^
Gender, male/female	10/8	7/5	3/3	1.00^‡^
Smokers, *n* (%)	4 (22.2)	3 (25.0)	1 (16.7)	1.00^‡^
Alcohol drinkers, *n* (%)	11 (61.1)	10 (83.3)	1 (16.7)	.01^‡^
BMI (kg/m^2^ [mean ± *SD*])	21.9 ± 2.3	22.1 ± 2.6	21.7 ± 2.0	.77^†^
HP-IgG (IU/ml [mean ± *SD*])	40.8 ± 22.6	42.3 ± 26.8	37.8 ± 12.0	.70^†^
Plasma total ghrelin	58.7 ± 32.6	72.3 ± 30.8	31.7 ± 14.4	.01^†^
Acyl ghrelin	11.7 ± 10.2	15.0 ± 11.1	5.2 ± 3.2	.05^†^
Pepsinogens				
PG I (ng/ml)	58.4 ± 34.7	63.0 ± 40.6	49.2 ± 17.7	.44^†^
PG II (ng/ml)	18.3 ± 10.2	17.9 ± 11.5	19.3 ± 7.8	.79^†^
PG I/II ratio	3.5 ± 1.3	3.8 ± 1.4	2.8 ± 1.0	.11^†^
Adiponectin	3.9 ± 1.9	3.8 ± 1.9	4.0 ± 1.9	.87^†^
HDL-C	66.4 ± 16.2	67.8 ± 14.2	63.7 ± 21.0	.63^†^
LDL-C	120.6 ± 22.7	116.6 ± 24.2	128.5 ± 19.0	.31^†^
TG	81.0 ± 40.4	69.9 ± 24.5	103.2 ± 58.0	.70^†^
Glucose	101.8 ± 11.2	104.1 ± 9.2	97.2 ± 14.3	.23^†^
HbA1c	5.5 ± 0.4	5.5 ± 0.5	5.6 ± 0.3	.91^†^

SD: standard deviation; BMI: body mass index; *H. pylori*: *Helicobacter pylori*; HP-IgG: *Helicobacter pylori* immunoglobulin G; PG: pepsinogen; HDL-C: high-density lipoprotein cholesterol; LDL-C: low-density lipoprotein cholesterol; TG: triglycerides; HbA1c: haemoglobin A1c.

Alcohol drinkers were defined as people who consumed at least one alcoholic drink per month.

^†^Student’s *t*-test.

^‡^Fisher’s exact test.

### *Changes in serum HP-IgG after* H. pylori *eradication*

Serum HP-IgG values decreased from before eradication to 48 weeks after eradication (*F*_1.13_ = 32.750, *p* < .01), but there was no significant difference between open- and closed-type atrophic gastritis (*F*_1_ = 0.091, *p* = .767) (Supplementary Figure 1).

### Comparison of the extent of gastric atrophy with plasma ghrelin and pepsinogen levels

**Figure 1.**
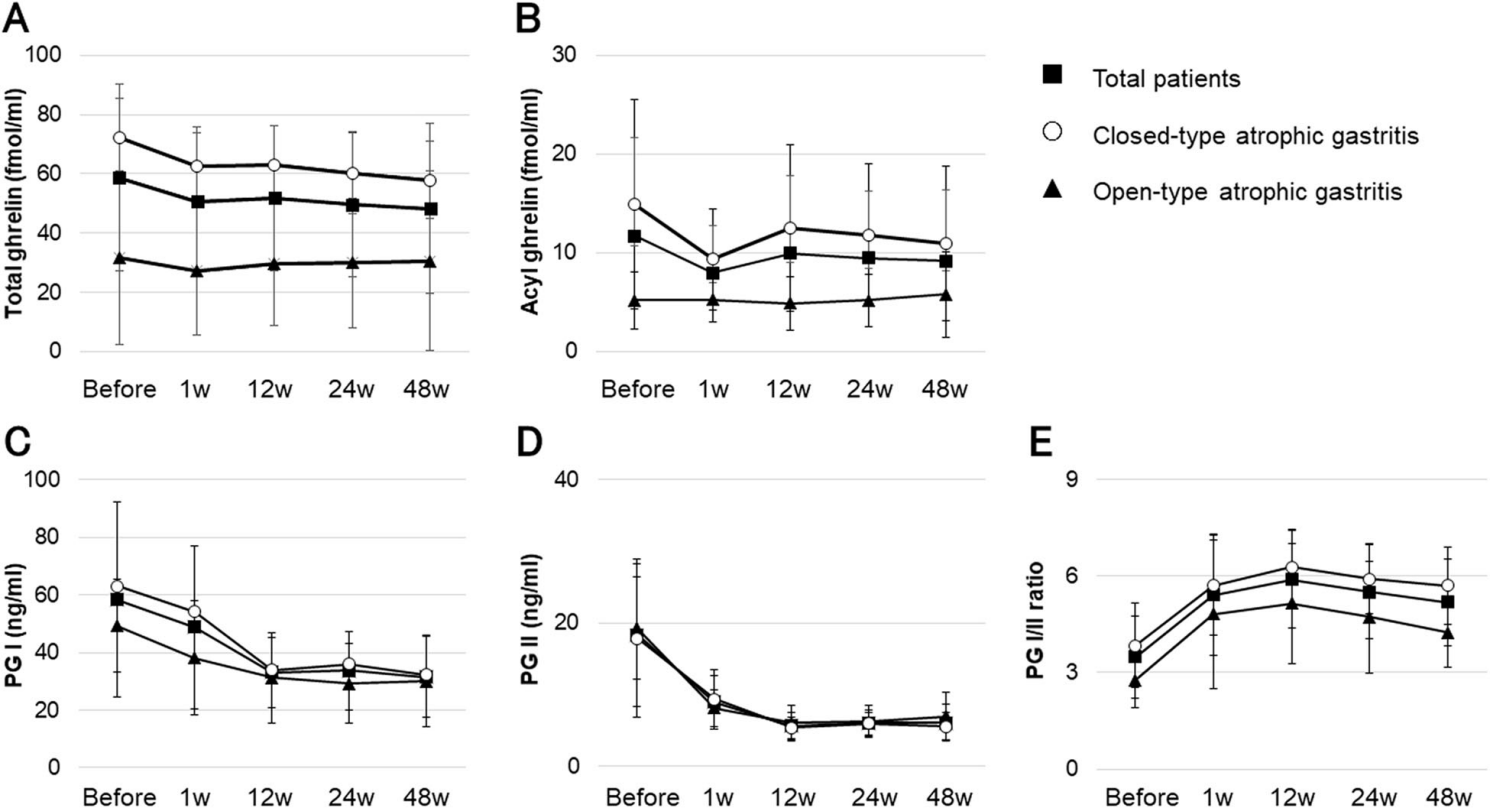
Plasma total and acyl ghrelin levels, serum PGI and II levels, and PG I/II ratios from before to 48 weeks after *H. pylori* eradication. (A,B) Total and acyl plasma ghrelin levels showed no significant change from before to 48 weeks after eradication (*F*_4_ = 0.885, *p* = .48; and *F*_4_ = 0.212, *p* = .93, respectively), but there was a significant difference between open-type and closed-type atrophic gastritis (*F*_1_ = 10.193, *p* < .01; and *F*_1_ = 7.844, *p* = .01, respectively). (C,D) PG I and PG II values decreased from before to 48 weeks after eradication (*F*_1.72_ = 10.510, *p* < .01; and *F*_0.11_ = 24.247, *p* < .01, respectively), but there was no significant difference between open-type and closed-type atrophic gastritis (*F*_1_ = 0.814, *p* = .38; and *F*_1_ = 0.039, *p* = .85, respectively). (E) The PG I/II ratio increased from before to 48 weeks after eradication (*F*_2.21_ = 18.310, *p* < .01), and there was a marginal significant difference between open-type and closed-type atrophic gastritis (*F*_1_ = 3.275, *p* = .09). PG: pepsinogen. Error bars represent the standard deviation.

Changes in total and acyl plasma ghrelin levels, in PG I and PG II levels, and in the PG I/II ratio before and up to 48 weeks after eradication are shown in Figure 1. Total plasma ghrelin levels did not change significantly from before to 48 weeks after eradication (*F*_4_ = 0.885, *p* = .48), but there was a significant difference in total plasma ghrelin levels between open-type and closed-type atrophic gastritis (*F*_1_ = 10.193, *p* < .01) (Figure 1(A)). Similarly, acyl plasma ghrelin levels showed no significant change from before to 48 weeks after eradication (*F*_4_ = 0.212, *p* = .93), but there was a significant difference in acyl plasma ghrelin levels between open-type and closed-type atrophic gastritis (*F*_1_ = 7.844, *p* = .01) (Figure 1(B)). PG I and PG II values decreased from before to 48 weeks after eradication (*F*_1.72_ = 10.510, *p* < .01 and *F*_0.11_ = 24.247, *p* < .01, respectively), but there was no significant difference of PG I and PG II values between open-type and closed-type atrophic gastritis (*F*_1_ = 0.814, *p* = .38 and *F*_1_ = 0.039, *p* = .85, respectively) (Figures 1(C,D)). The PG I/II ratio increased from before to 48 weeks after eradication (*F*_2.21_ = 18.310, *p* < .01), and there was a marginally significant difference between open-type and closed-type atrophic gastritis (*F*_1_ = 3.275, *p* = .09) (Figure 1(E)). PG II values and the PG I/II ratio before eradication were associated with an increase in the PGI/II ratio (*r* = 0.614, *p* < .01 and *r* = −0.639, *p* < .01, respectively).

**Figure 2.**
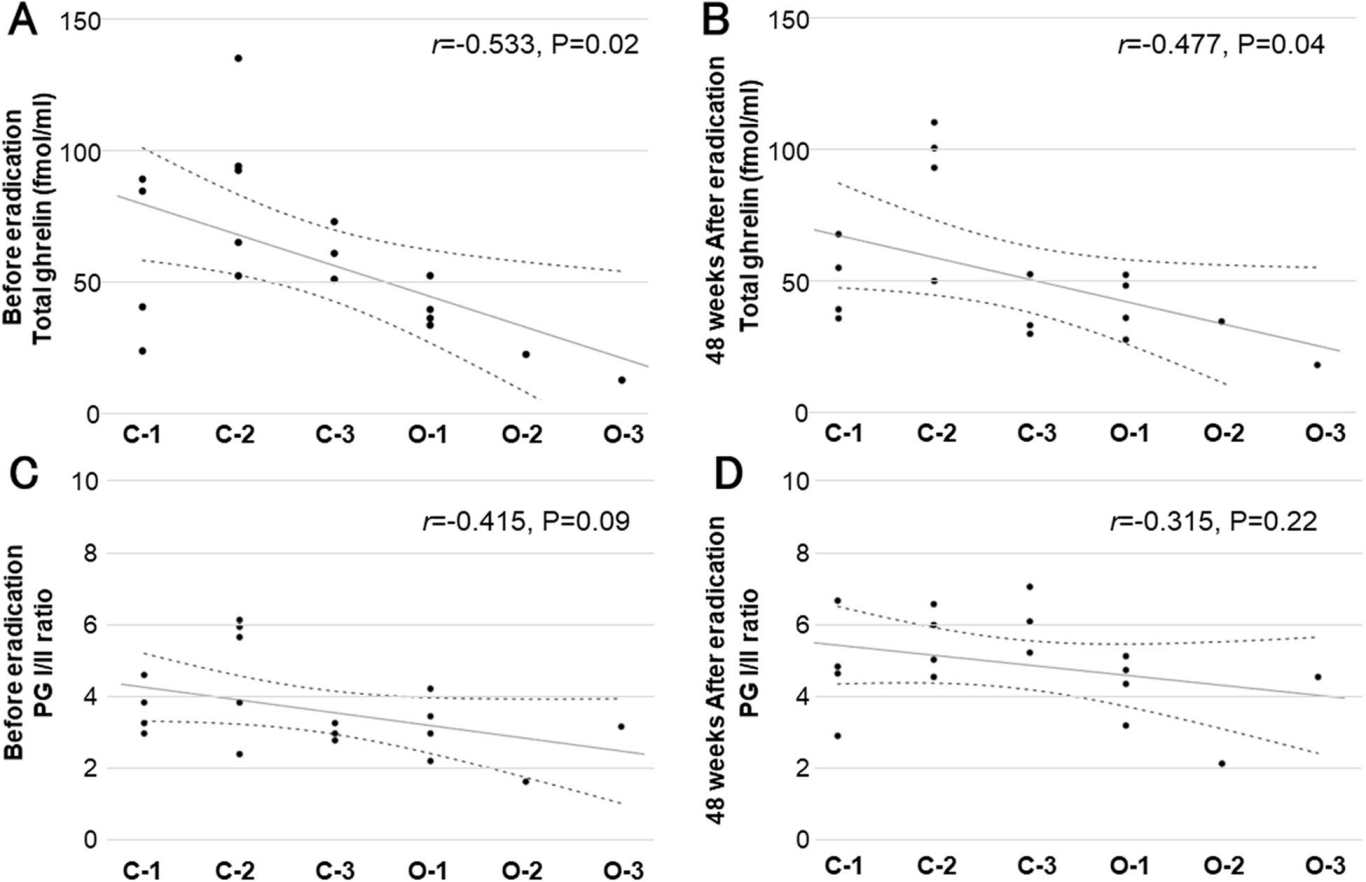
Distribution of plasma ghrelin levels and PG I/II ratios from before to 48 weeks after *H. pylori* eradication and endoscopic atrophy progression. (A) Plasma total ghrelin levels were significantly inversely correlated to the development of atrophic gastritis (*r* = −0.533, *p* = .02) (B) This correlation was maintained even at 48 weeks after eradication (*r* = −0.477, *p* = .04). (C) PG I/II ratios were marginally significantly inversely correlated with the development of atrophic gastritis (*r* = −0.415, *p* = .09). (D) This correlation was no longer significant at 48 weeks after eradication (*r* = −0.315, *p* = .22). PG: pepsinogen. Solid lines depict the regression line of the data, and dotted lines represent the 95% confidence interval.

Sub-divisional patterns of the Kimura–Takemoto classification, the distribution of the total ghrelin levels, and the PG I/II ratio before and at 48 weeks after eradication are shown in Figure 2. Total plasma ghrelin levels were significantly and inversely correlated with the development of atrophic gastritis (*r* = −0.533, *p* = .02) (Figure 2(A)). This correlation was maintained even at 48 weeks after eradication (*r* = −0.477, *p* = .04) (Figure 2(B)). The PG I/II ratio showed a marginally significant negative correlation with the development of atrophic gastritis (*r* = −0.415, *p* = .09) (Figure 2(C)). This correlation was no longer significant at 48 weeks after eradication (*r* = −0.315, *p* = .22) (Figure 2(D)).

### Comparison of the severity of histological gastric mucosal atrophy and intestinal metaplasia with plasma ghrelin and pepsinogen levels

**Figure 3.**
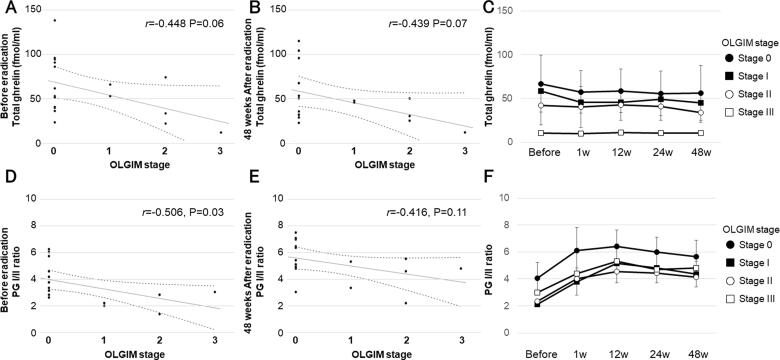
Plasma ghrelin levels and PG I/II ratios from before to 48 weeks after *H. pylori* eradication and histological intestinal metaplasia progression. (A,B) Plasma total ghrelin levels were marginally significantly correlated with OLGIM stage before and 48 weeks after *H. pylori* eradication (*r* = −0.448, *p* = .06; and *r* = −0.439, *p* = .07, respectively). (C) Total plasma ghrelin levels demonstrated no significant change from before to 48 weeks after eradication (*F*_4_ = 0.380, *p* = .82), and there was no significant difference between OLGIM stage (*F*_3_ = 1.432, *p* = .28). (D,E) PG I/II ratios correlated significantly with OLGIM stage before *H. pylori* eradication, but not at 48 weeks after *H. pylori* eradication. (*r* = −0.506, *p* = .03; and *r* = −0.416, *p* = .11, respectively). (F) The PG I/II ratio increased from before to 48 weeks after eradication (*F*_2.28_ = 9.310, *p* < .01), and there was no significant difference between OLGIM stages (*F*_3_ = 2.430, *p* = .11). PG: pepsinogen; OLGIM: operative link on gastric intestinal metaplasia. Solid lines depict the regression line of the data, and dotted lines represent the 95% confidence interval (A,B,D,E). Error bars represent the standard deviation (C, F).

The severity of histological gastric mucosal intestinal metaplasia was assessed using the OLGIM. The OLGIM stages, the distribution of the total ghrelin levels, and the PG I/II ratio before eradication are shown in Figure 3. The plasma total ghrelin levels had a marginally significant negative correlation with the OLGIM stage before eradication and 48 weeks after eradication (*r* = −0.448, *p* = .06 and *r* = −0.439, *p* = .07, respectively) (Figures 3(A,B)). Total plasma ghrelin levels showed no significant change from before eradication to 48 weeks (*F*_4_ = 0.380, *p* = .82), and there was no significant difference between the groups with respect to the OLGIM stage (*F*_3_ = 1.432, *p* = .28) (Figure 3(C)). The PG I/II ratio correlated significantly with the OLGIM stage before eradication (*r* = −0.506, *p* = .03) (Figure 3(D)), but there was no significant difference in the OLGIM stage (*r* = −0.416, *p* = .11) (Figure 3(E)). The PG I/II ratio increased from before to 48 weeks after eradication (*F*_2.28_ = 9.310, *p* < .01), and there was no significant difference between the groups with respect to the OLGIM stage (*F*_3_ = 2.430, *p* = .11) (Figure 3(F)). Analysis of the correlation between the OLGIM score and development of endoscopic gastric mucosal atrophy showed that the OLGIM scores for open-type and closed-type gastritis were 1.33 ± 1.21 and 0.25 ± 0.62, respectively, and were significantly higher for open-type gastritis (*p* = .02). Furthermore, a linear analysis of the subscores of the Kimura–Takemoto classification (from C-1 to O-3) and the OLGIM scores revealed a significant correlation (*p* < .01).

The severity of histological gastric mucosal atrophy was assessed using the OLGA. The OLGA stages, the distribution of total ghrelin levels, and the PG I/II ratios before eradication are shown in Supplementary Figure 2. Fifteen patients were classified as stage III, two as stage II, and one as stage I. As most cases were classified as stage III, the association between the OLGA stage and these markers was considered uninterpretable.

### *Trends in lipid metabolism, carbohydrate metabolism, and hormones after* H. pylori *eradication*

**Figure 4.**
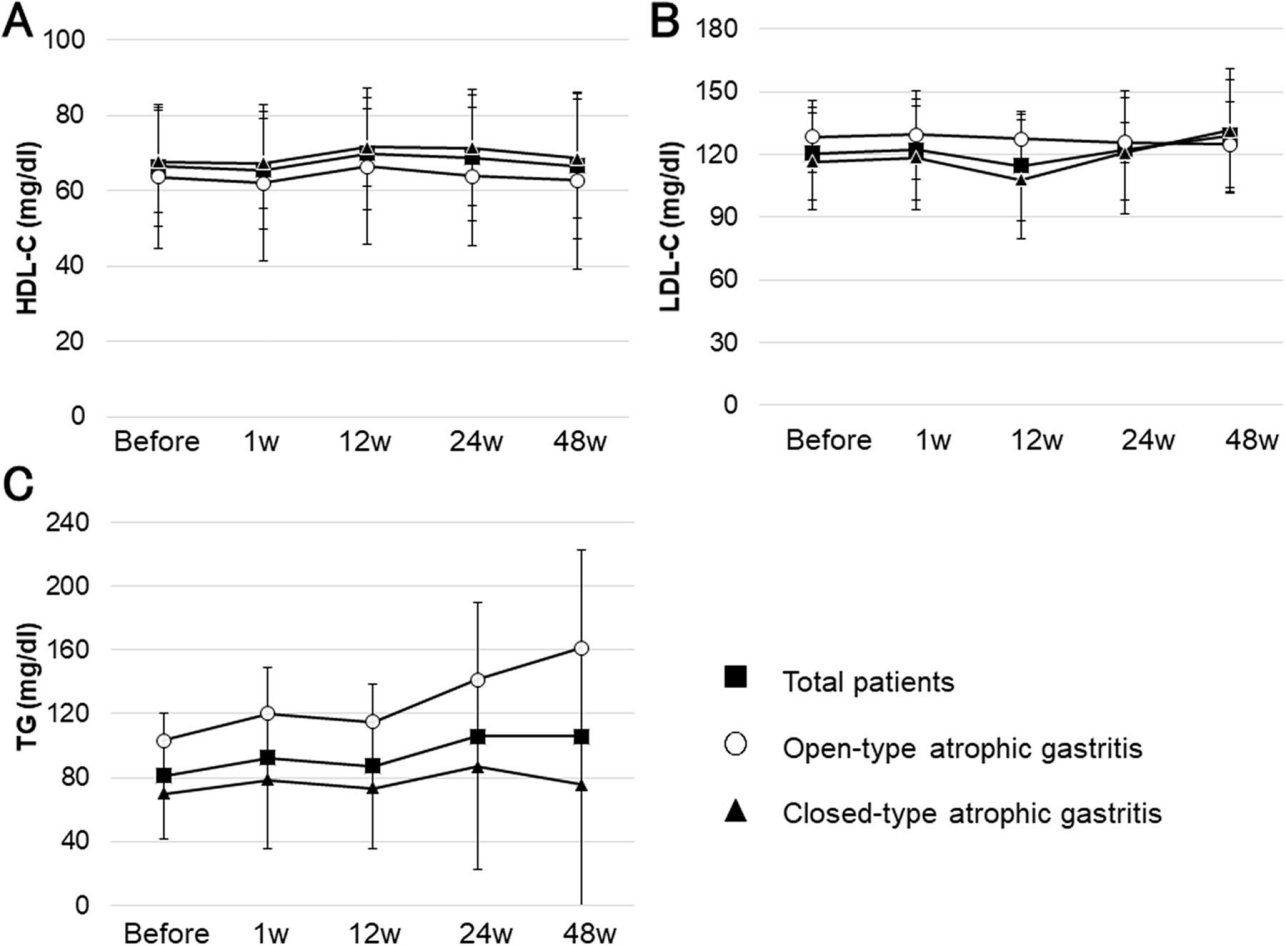
Lipid marker levels from before to 48 weeks after *H. pylori* eradication. HDL-C, LDL-C, and TG levels demonstrated no significant change from before to 48 weeks after eradication (*F*_2.50_ = 1.148, *p* = .34; *F*_4_ = 1.256, *p* = .30; *F*_1.32_ = 1.343, *p* = .27, respectively), and there was no significant difference in any of these markers between open-type and closed-type atrophic gastritis (*F*_1_ = 0.497, *p* = .49; *F*_1_ = 0.355, *p* = .56; *F*_1_ = 2.486, *p* = .14, respectively). HDL-C: high-density lipoprotein cholesterol; LDL-C: low-density lipoprotein cholesterol; TG: triglycerides. Error bars represent the standard deviation.

Trends in lipid markers from before to 48 weeks after eradication are shown in Figure 4. HDL-C, LDL-C, and TG levels did not change significantly from before eradication to 48 weeks after eradication (*F*_2.50_ = 1.148, *p* = .34; *F*_4_ = 1.256, *p* = .30; and *F*_1.32_ = 1.343, *p* = .27, respectively), and there was no significant difference in any of the markers between open-type and closed-type atrophic gastritis (*F*_1_ = 0.497, *p* = .49; *F*_1_ = 0.355, *p* = .56; and *F*_1_ = 2.486, *p* = .14, respectively). Regarding trends in TG values, there seemed to be an increase in the open-type gastritis group, but this was due to a marked increase in the TG level in one subject (from 127 mg/dl before eradication to 548 mg/dl at 1 year after eradication). The mean TG value for the other 17 subjects was 78.5 ± 41.3 mg/dl before eradication and 78.3 ± 40.4 mg/dl at 1 year after eradication, i.e. little change.

**Figure 5.**
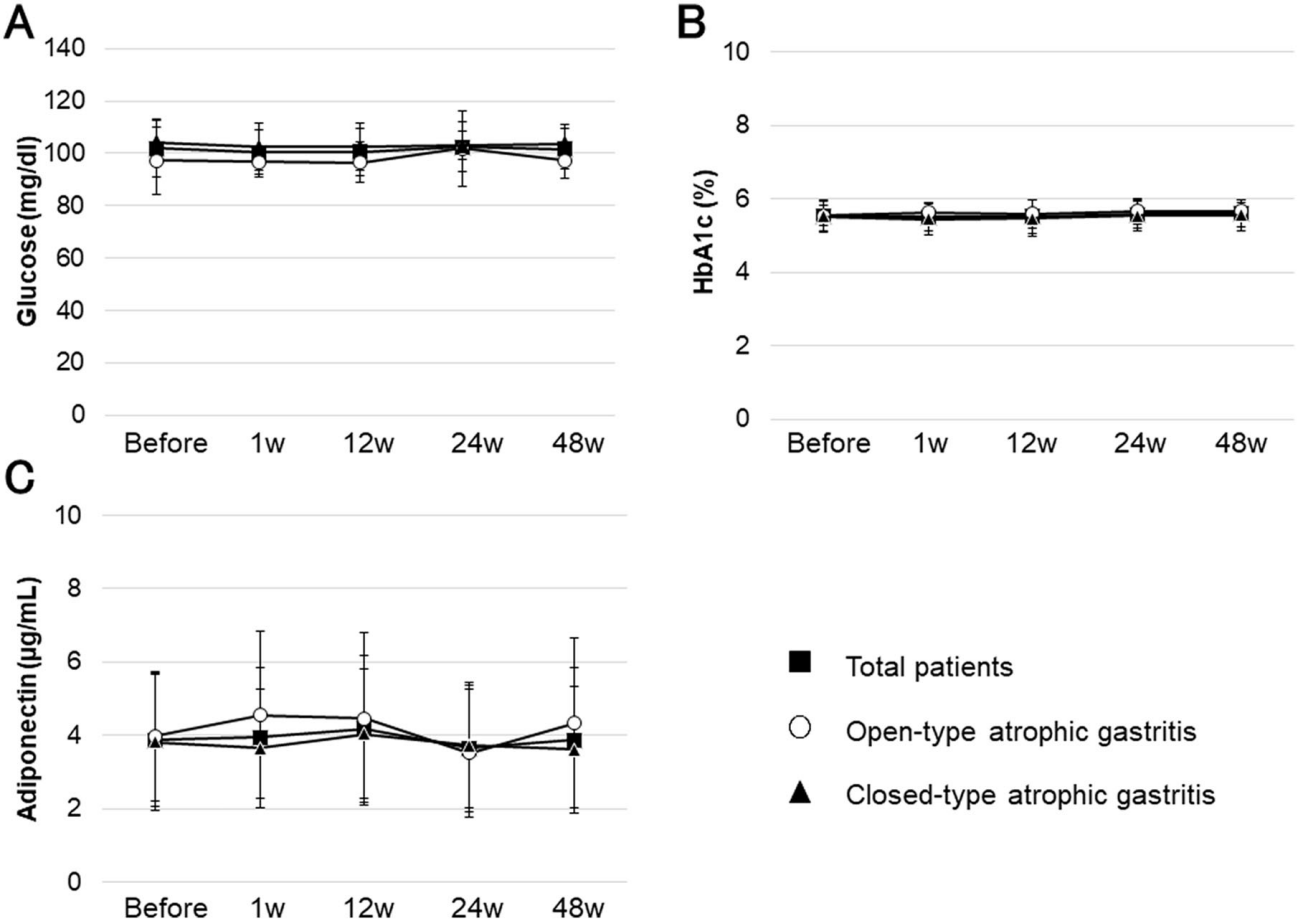
Carbohydrate marker levels from before to 48 weeks after *H. pylori* eradication. Glucose, HbA1c, and adiponectin levels showed no significant change from before to 48 weeks after eradication (*F*_4_ = 0.452, *p* = .77; *F*_4_ = 0.797, *p* = .53; *F*_4_ = 2.642, *p* = .07, respectively), and there was no significant difference in any of these markers between open-type and closed-type atrophic gastritis (*F*_1_ = 2.287, *p* = .15; *F*_1_ = 0.146, *p* = .71; *F*_1_ = 0.154, *p* = .70, respectively). HbA1c: haemoglobin A1c. Error bars represent the standard deviation.

Trends in carbohydrate markers from before eradication to 48 weeks after eradication are shown in Figure 5. Glucose, HbA1c, and adiponectin levels did not change significantly from before to 48 weeks after eradication (*F*_4_ = 0.452, *p* = .77; *F*_4_ = 0.797, *p* = .53; and *F*_4_ = 2.642, *p* = .07, respectively), and there was no significant difference in any of these markers between open-type and closed-type atrophic gastritis (*F*_1_ = 2.287, *p* = .15; *F*_1_ = 0.146, *p* = .71; and *F*_1_ = 0.154, *p* = .70, respectively).

## Discussion

The strength of this study was identifying the relationship between endoscopic and histological gastric mucosal atrophy and intestinal metaplasia, plasma total ghrelin and acyl ghrelin levels, and serum PG levels through regular monitoring from before *H. pylori* eradication to 1 year after eradication. There were clear differences in total plasma and acyl ghrelin levels between closed-type and open-type endoscopic atrophic gastritis before *H. pylori* eradication, and this difference was maintained for up to 1 year after *H. pylori* eradication. This means that total plasma and acyl ghrelin levels reflect the extent of endoscopically determined atrophic changes over the long term after *H. pylori* eradication. By contrast, PG I and PG II showed a clear decrease after *H. pylori* eradication, whereas the PG I/II ratio increased significantly, reflecting the relatively large decrease in PG II. As shown in Figures 1(D,E), the PG I/II ratio for both closed- and open-type gastritis converged at almost the same level, suggesting that it will be difficult to use this parameter to estimate the risk of gastric cancer caused by gastric mucosal atrophy after *H. pylori* eradication [31].

Several studies have compared ghrelin levels after *H. pylori* eradication [15,32–38]. Depending on the age or underlying condition of the subject, ghrelin levels either increase, decrease, or remain unchanged, and there is still no clear consensus. For example, a study in children showed an increase in total ghrelin levels after *H. pylori* eradication, while another study showed that acyl ghrelin levels decreased in patients with dyspepsia [32,33]. The results of the present study might be more generalized than those of other studies because we did not target a specific type of disease. Previously, we reported that plasma ghrelin levels reflect the degree of atrophy after *H. pylori* eradication, similar to the current study [15], whereas other previous studies did not measure ghrelin levels at a defined time point after *H. pylori* eradication. The strength of the present study is the assessment of ghrelin levels over time (up to 48 weeks) in a set number of subjects.

We also found that the OLGIM score, which indicates the degree of histological intestinal metaplasia, correlated inversely with plasma ghrelin levels from before to 48 weeks after *H. pylori* eradication. Originally, the OLGIM was developed as a marker of gastric cancer risk (rather than the OLGA). In this study, the OLGIM score correlated with the degree of endoscopic gastric mucosal atrophy, confirming the usefulness of the OLGIM score as a marker of gastric cancer risk. The OLGIM system was developed because the severity of atrophic gastritis remains a difficult histopathologic diagnosis, with a low interobserver agreement, whereas intestinal metaplasia is associated with the high interobserver agreement [28]. Some studies report that OLGIM is a histological marker of gastric cancer risk [39–41].

There was little change in lipid markers after *H. pylori* eradication therapy, except for a marked increase in serum TG levels in one subject (Figure 4). Despite various reports of an association between *H. pylori* and dyslipidemia, no specific conclusion has been reached. Patel et al. reported that serum total cholesterol and serum TG did not differ according to the presence or absence of *H. pylori* infection [42]. A study of 1650 subjects by Takashima et al. reported that HDL-C levels were significantly lower in patients with *H. pylori* infection [43]. Gunji et al. reported that *H. pylori* infection is an independent risk factor that affects HDL-C and LDL-C levels, and promotes the development and progression of atherosclerosis [44]. The results of the present study suggest that associations between lipid markers and *H. pylori* eradication cannot be ruled out, despite the finding that most subjects showed no change in lipid markers after *H. pylori* eradication; the large TG changes in one case may be due to a specific predisposition. Recent reports suggest an association between the gut microbiota and dyslipidemia; therefore, future studies should focus on specific patients whose lipid metabolism was affected before and after eradication [45,46].

There was little variation in the glucose metabolic markers before and after eradication in our study subjects (Figure 5). Regarding the association between *H. pylori* infection and diabetes, it was reported that the rate of *H. pylori* infection is significantly higher in patients with type 2 diabetes; that *H. pylori* infection increases the risk of developing diabetes; and that diabetic patient without *H. pylori* infection have significantly lower HbA1c levels than diabetic patients with *H. pylori* infection [47–50]. Regarding the association between *H. pylori* eradication and diabetes, Dai et al. reported that *H. pylori* eradication does not improve glycemic control in patients with type 2 diabetes [50]. In the present study, which did not include diabetic patients, there were no obvious changes in blood glucose levels.

A clear limitation of this study is that it was a non-randomized trial. First, it did not include follow-up subjects without *H. pylori* eradication; therefore, it is not possible to assess the extent to which plasma ghrelin and serum PG levels change naturally over time. Second, the study assessed outcomes before and up to ∼1 year after *H. pylori* eradication but did not assess data for longer periods. Third, this study was a pilot study with a small number of patients. It is possible that a study with a larger cohort will provide new insight into the data that were not significantly different in this study.

In conclusion, plasma levels of ghrelin correlate well with gastric mucosal atrophy, even after *H. pylori* eradication.

## Supplementary Material

Supplemental Material

## Data Availability

The data used for the current study are accessible from the corresponding authors upon reasonable request.
