# Supplementary figures and images for: Development of plasma ghrelin level as a novel marker for gastric mucosal atrophy after Helicobacter pylori eradication
Source: Ann Med. 2022 Jan 10;54(1):170–80. doi: 10.1080/07853890.2021.2024875 (PMC9891226; doi:10.1080/07853890.2021.2024875)

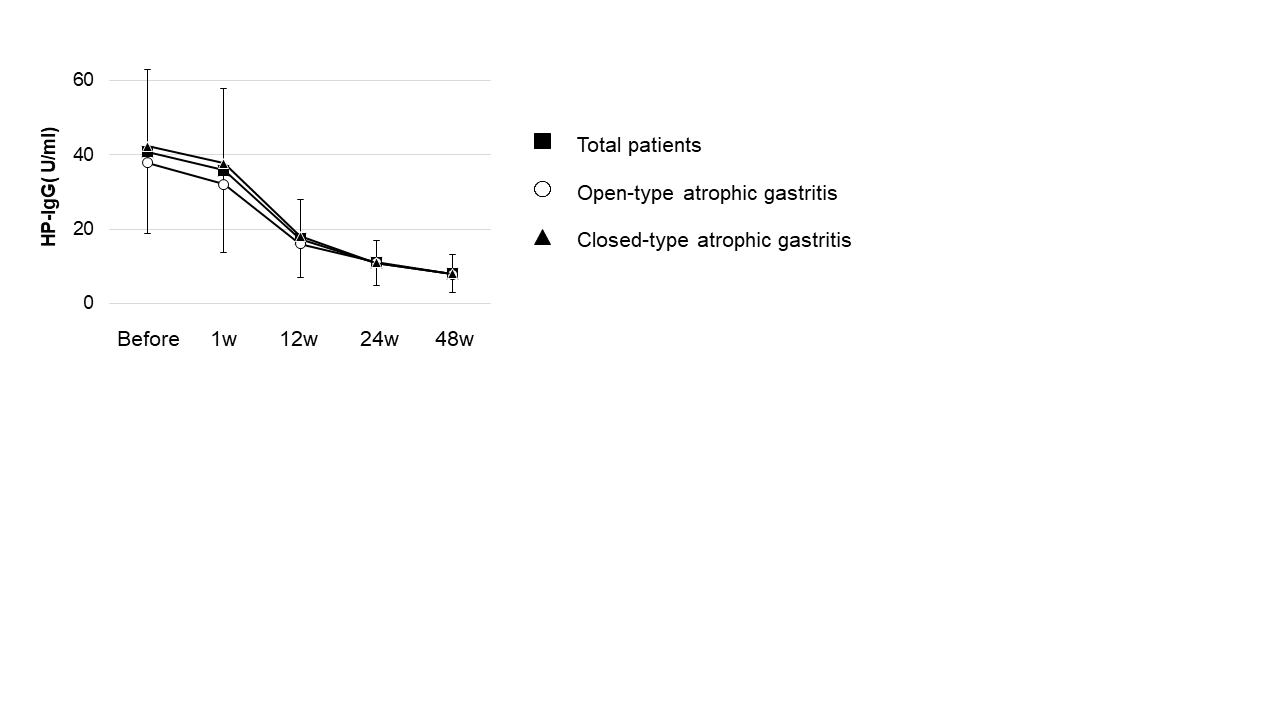

Supplement: Supplemental Material [file IANN_A_2024875_SM2883.zip › Supplemental figures/Supplementary Figure 1.tif]

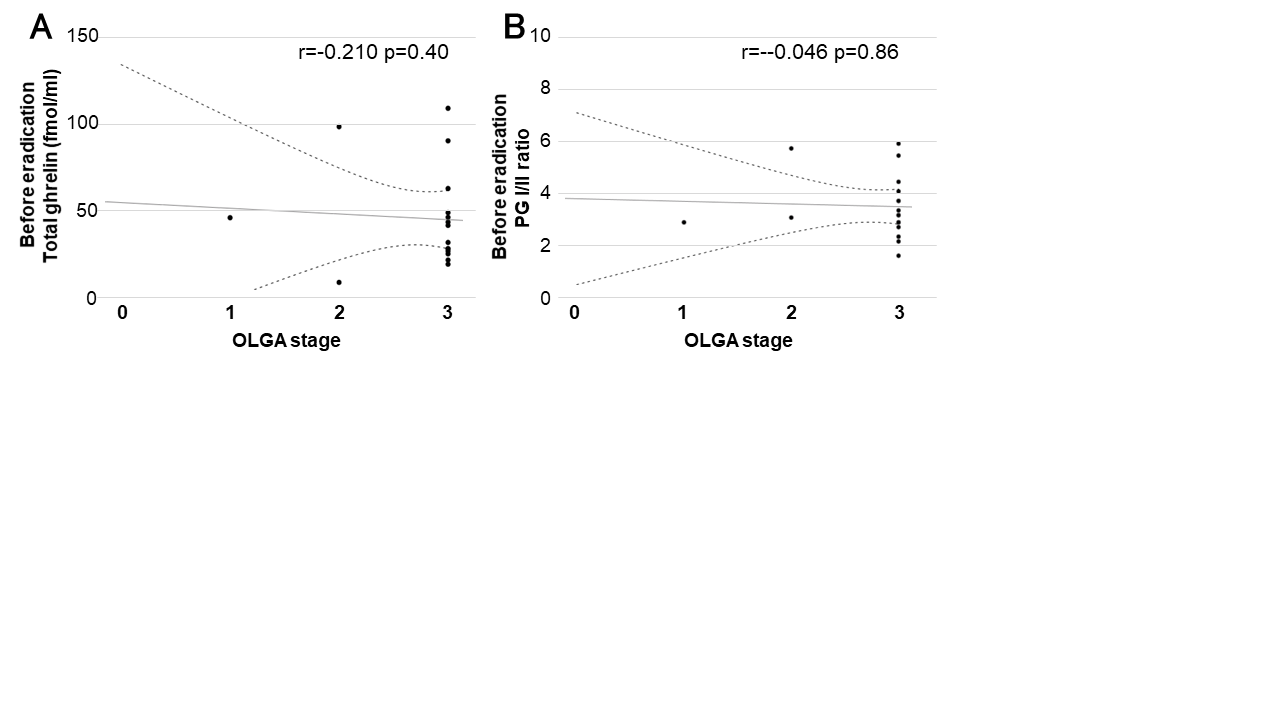

Supplement: Supplemental Material [file IANN_A_2024875_SM2883.zip › Supplemental figures/Supplementary Figure 2.tif]
